# The impact of social deprivation on mortality following acute myocardial infarction, stroke or subarachnoid haemorrhage: A record linkage study

**DOI:** 10.1186/s12872-015-0045-x

**Published:** 2015-07-18

**Authors:** Kymberley Thorne, John G. Williams, Ashley Akbari, Stephen E. Roberts

**Affiliations:** College of Medicine, Swansea University, Singleton Park, Swansea, SA2 8PP UK

**Keywords:** Mortality, Social deprivation, Risk factors, Acute myocardial infarction, Stroke, Subarachnoid haemorrhage

## Abstract

**Background:**

The impact of social deprivation on mortality following acute myocardial infarction (AMI), stroke and subarachnoid haemorrhage (SAH) is unclear. Our objectives were, firstly, to determine, for each condition, whether there was higher mortality following admission according to social deprivation and secondly, to determine how any higher mortality for deprived groups may be correlated with factors including patient demographics, timing of admission and hospital size.

**Methods:**

Routinely collected, linked hospital inpatient, mortality and primary care data were analysed for patients admitted as an emergency to hospitals in Wales between 2004 and 2011 with AMI (*n* = 30,663), stroke (37,888) and SAH (1753). Logistic regression with Bonferroni correction was used to examine, firstly, any significant increases in mortality with social deprivation quintile and, secondly, the influence of patient demographics, timing of admission and hospital characteristics on any higher mortality among the most socially deprived groups.

**Results:**

Mortality was 14.3 % at 30 days for AMI, 21.4 % for stroke and 35.6 % for SAH. Social deprivation was significantly associated with higher mortality for AMI (25 %; 95 % CI = 12 %, 40 %) higher for quintile V compared with I), stroke (24 %; 14 %, 34 %), and non-significantly for SAH (32 %; −7 %, 87 %).

The higher mortality at 30 days with increased social deprivation varied significantly according to patient age for AMI patients and time period for SAH. It was also highest for both AMI and stroke patients, although not significantly for female patients, for admissions on weekdays and during autumn months.

**Conclusions:**

We have demonstrated a positive association between social deprivation and higher mortality following emergency admissions for both AMI and stroke. The study findings also suggest that the influence of patient demographics, timing of admission and hospital size on social inequalities in mortality are quite similar for AMI and stroke.

**Electronic supplementary material:**

The online version of this article (doi:10.1186/s12872-015-0045-x) contains supplementary material, which is available to authorized users.

## Background

Acute myocardial infarction (AMI), stroke and subarachnoid haemorrhage (SAH) are all associated with high mortality following hospitalisation [1]. Some research suggests that mortality rates are significantly higher for more deprived than more affluent patients following admission for cardiovascular disease [2–6], stroke [3, 7–13] and SAH [8, 14]. Possible reasons include patient comorbidities, poor diet, obesity and other lifestyle risk factors, as well as differences in health seeking behaviours and inequalities in access to and compliance with treatment and care. However, other studies have reported either no significant association with mortality for cardiovascular diseases [15–17] and stroke [18–20], or that mortality was lower with increased social deprivation for cardiovascular conditions [21, 22] and stroke [23].

There is a lack of evidence for the reasons why social deprivation inequalities in mortality following admission for AMI, stroke or SAH may be affected by factors including patient demographics, timing of admission and the size of the hospital. It is possible that day of the week, year of admission and hospital size could act as possible mediators of the relationship between social deprivation and 30 day mortality for AMI, stroke and SAH. If so, we hypothesise that deprived cases would have a worse prognosis if admitted on weekdays and public holidays as any social inequalities in access to urgent investigative and therapeutic services and surgery may be widened by reduced operational levels of services at weekends. Additionally, it has been reported that affluent patients get to hospital sooner following a stroke [9] which may be linked to the admitting hospital’s location and size.

In order to explore these possibilities, we investigated associations between social deprivation and mortality following AMI, stroke and SAH in a large population in the UK. The first objective of this study was to determine, for each condition, whether there was higher mortality at 30 days following admission according to social deprivation. Secondly, we determined how any higher mortality for deprived groups may be correlated with factors such as patient demographics, timing of admission and hospital size.

## Methods

### Study design

To address these study objectives, we used systematic record linkage of national inpatient, mortality and primary care data across Wales (population 3 million). All records were held within the Secure Anonymised Information Linkage (SAIL) Databank, which includes the Patient Episode Database for Wales (PEDW) and the Welsh Administrative Register (AR). All records were linked using a unique anonymised linking field (ALF) for each patient based primarily on the patient’s National Health Service (NHS) number and secondly, on other anonymised patient identifiers such as date of birth, gender, postcode and first name and surname by applying a probabilistic matching algorithm MACRAL (Matching Algorithm for Consistent Results in Anonymised Linkage). More details on the SAIL databank and the MACRAL methodology can be found in articles by Lyons et al. and Ford et al. to confirm the accuracy of linkage using this method [24, 25].

To identify all deaths that occurred following discharge from hospital as well as in hospital, the inpatient data were systematically linked to death certificate data from the Office for National Statistics (ONS) and the Welsh AR. Linkage was possible for 100 % of patients in our dataset.

To obtain information on comorbidities recorded from primary care consultations as well as inpatient admissions over the previous 5 years, the inpatient data were also linked to SAIL national primary care data. The SAIL databank has access to 40 % of GP practice data in Wales (approximately 1 million records). We also adjusted for whether each patient had a previous GP or inpatient visit been to eliminate any bias during logistic regression modelling.

### Ethics statement

Ethical approval and patient consent for the study data was not required as we were using pseudo-anonymised data. Study approval was obtained instead from the Information Governance Review Panel, which is represented by the National Research Ethics Service, the British Medical Association Ethics Advisor, the Caldicott Guardians and NHS Wales Informatics Service. The linked study data sources we used are publicly available to other researchers.

### Inclusion and exclusion criteria

We selected all emergency admissions to Welsh hospitals where AMI, stroke or SAH were recorded as the principal diagnosis on the discharge record. The International Classification of Diseases tenth revision (ICD-10) codes used for the three conditions were I21, I61–64 and I60 respectively. We included patients aged 18 years or over, admitted between January 1^st^ 2004 and December 31^st^ 2011 and followed them up for 12 months. Admissions were excluded if they were not emergencies (e.g. elective) or if they occurred within 365 days of a previous admission’s discharge date.

### Mortality

Mortality rates at 30 days following the admission were used as the primary outcome measure and at 7 days and 365 days as secondary outcome measures. Thirty day mortality is a common outcome measure in the literature. Mortality was based on all causes of death. We included deaths occurring during the inpatient stay and those occurring following discharge.

### Social deprivation

Social deprivation for the Welsh population was measured using the Welsh Index of Multiple Deprivation (WIMD) 2008 [26], produced by the Welsh Assembly Government. It is an area-based measure consisting of seven separate domains of deprivation: ‘income’ (23.5 % contribution), ‘employment’ (23.5 %), ‘health’ (14 %), ‘education’ (14 %), ‘access to services’ (10 %), ‘housing’ (5 %), ‘physical environment’ (5 %) and ‘community safety’ (5 %). It is based on 1896 Lower Super Output Areas (average population = ~1500 each) and provides a deprivation score which was ranked and assigned to one of five deprivation quintiles (I = least deprived and V = most deprived quintile).

### Risk factors

We assessed the following key risk factors to determine whether they significantly mediated the relationship between social deprivation and mortality at 30 days following admission, comparing the least and most deprived cases, using the least deprived quintile as the reference group.

#### Patient demographics

The patient’s age on admission was collected for each case. Age was grouped into “<65 years”, “65 to 74 years”, “75 to 84 years” and “85+ years” The patient’s gender was also recorded.

#### Timing of admission

We investigated any impact of the day of admission on mortality by assigning weekdays (Monday 00:00 to Friday 23:59), weekends (Saturday 00:00 to Sunday 23:59) and public holiday (8 per year) with public holidays prioritised over weekdays and weekends in this classification. We also investigated the season of admission (winter = Dec to Feb; spring = Mar to May; summer = Jun to Aug; autumn = Sept to Nov) and grouped year of admission (“2004–2005”, “2006–2008” and “2009–2011”).

#### Hospital size

Hospital size at the time of admission was included with the following categories: “Small District General Hospital (DGH) (100–399 beds)”, “medium DGH (400–599 beds)”, “large DGH (600+ beds)” and “community hospital (less than 100 beds)”.

### Patient comorbidities

When investigating mortality, we also adjusted for age group, gender and comorbidities. Specifically, we adjusted for any impact of the following 11 major patient comorbidities recorded in any diagnostic position during the admission, or within the previous 5 years from primary and inpatient care records where available: ischaemic heart disease (I20–I25) or other cardiovascular diseases (I00–I15, I26–I52), cerebrovascular disease (I60–I69), other circulatory diseases (I70–I99), malignancies (C00–C97), chronic obstructive pulmonary disease (J40–J44), asthma (J45–J46), diabetes (E10–E14), dementia (F00–F03, F05.1, G30), liver disease (K70–K77) and renal failure (N17–N19) [27].

### Sample size calculations

Detecting a 30 % increased risk in adjusted mortality for quintile V compared to quintile I based on mortality rates of 35, 15 and 20 % for AMI, stroke and SAH respectively and using 80 % power and 5 % significance, would require 1150 AMI cases, 805 stroke cases and 360 SAH cases in each quintile.

### Methods of analysis

The main study outcome measures were percentage mortality rates and odds of mortality for quintile V versus quintile I at 30 days following admission for each condition. Secondary outcome measures were mortality rates and odds of mortality at 7 and 365 days.

Logistic regression was undertaken to establish, firstly, any increased odds of mortality associated with social deprivation at 7 and 30 days after admission. Secondly, it was used to establish how any higher mortality for deprived groups may be related to key risk factors including patient demographics, timing of admission and hospital size. To do this, we compared mortality in the least and most deprived quintiles, using the least deprived quintile as the reference category, for each individual strata in the risk factors; patient age group, sex, week day, season and time period of admission and hospital size. Thirdly, logistic regression was used to test for any interaction effects on mortality between social deprivation and, respectively, each of the study risk factors.

All logistic regression analyses adjusted for age, gender and the eleven patient comorbidities. To eliminate possible biases in the determination of patient co-morbidities from primary and secondary care diagnoses over the preceding 5 years, we also adjusted for patients with no previous inpatient admissions or primary care consultations. The logistic regression mortality odds ratios were presented with 95 % confidence intervals. Significance was measured at the conventional 5 % level.

There were no missing data for any factor other than patient gender and social deprivation (WIMD). For gender, one case admitted with AMI was missing. For WIMD, data was not available for the patients who were not normally resident in Wales. This equated to 3.2 % of AMI cases, 2.4 % of stroke cases and 3.1 % of SAH cases.

A Bonferroni correction was applied to account for multiple statistical tests for each condition. Results were displayed in tables to indicate whether they were significant before and after the correction was applied, although the text only refers to results significant after the correction was applied.

## Results

There were 30,663 cases of AMI, 37,888 cases of stroke and 1753 cases of SAH admitted as emergencies between January 2004 and December 2011. The mean age at admission was 70.9 years ± 13.8 for AMI, 75.7 years ± 12.7 for stroke and 60.7 years ± 16.0 for SAH. Males accounted for 60.2 %, 47.3 % and 36.5 % of cases respectively.

### Mortality and social deprivation

Mortality at 30 days was 14.3 % following admission for AMI, 21.4 % for stroke and 35.6 % for SAH (see Table 1). Corresponding mortality at 7 days for AMI, stroke and SAH was 9.2, 11.6 and 26.9 % and at 365 days it was 24.6, 37.7 and 40.4 % respectively.

**Table 1:** Admission numbers, mortality rates and adjusted odds ratios at 7, 30 and 365 days following hospitalisation, 2004–2012

Condition	Risk factor	Admissions (n)	7 days mortality rate	7 days OR (95 % CI)^b^	30 days mortality rate	30 days OR (95 % CI)^b^	365 days mortality rate	365 days OR (95 % CI)^b^
**AMI**	**All cases**	30,663	9.2 %	-	14.3 %	-	24.6 %	-
	**Age**							
	< 65y	9827	2.8 %	-	4.0 %	-	6.2 %	-
	65–74y	7088	7.4 %	-	10.6 %	-	17.2 %	-
	75–84y	8382	12.7 %	-	19.6 %	-	34.3 %	-
	85y+	5366	17.7 %	-	29.5 %	-	52.8 %	-
	**Gender**							
	Male	18,456	7.7 %	Ref	11.9 %	Ref	20.5 %	Ref
	Female	12,206	11.4 %	**1.09 (1.00, 1.18)**	17.8 %	1.07 (0.99, 1.15)	30.8 %	**1.10 (1.03, 1.17)**
	**Social deprivation**							
	I (Least deprived)	4776	8.9 %	Ref	14.5 %	Ref	25.3 %	Ref
	II	5527	8.7 %	1.04 (0.90, 1.19)	13.4 %	0.97 (0.86, 1.09)	23.6 %	0.98 (0.89, 1.09)
	III	6579	9.1 %	1.11 (0.97, 1.27)	14.3 %	1.07 (0.96, 1.20)	25.4 %	1.12 (1.01, 1.23)
	IV	6460	9.6 %	**1.23 (1.07, 1.40)**	15.0 %	**1.17 (1.05, 1.31)**	25.3 %	**1.13 (1.02, 1.24)**
	V (Most deprived)	6342	9.9 %	**1.34** ^**a**^ **(1.17, 1.53)**	15.0 %	**1.25** ^**a**^ **(1.12, 1.40)**	25.3 %	**1.20** ^**a**^ **(1.08, 1.32)**
**Stroke**	**All cases**	37,888	11.6 %	-	21.4 %	-	37.7 %	-
	**Age**							
	< 65y	6779	7.9 %	-	10.9 %	-	5.9 %	-
	65–74y	7989	9.4 %	-	14.9 %	-	25.1 %	-
	75–84y	13,171	11.7 %	-	21.6 %	-	39.6 %	-
	85y+	9949	15.7 %	-	33.4 %	-	60.4 %	-
	**Gender**							
	Male	17,936	9.8 %	Ref	17.6 %	Ref	32.1 %	Ref
	Female	19,952	13.1 %	**1.25** ^**a**^ **(1.16, 1.33)**	24.7 %	**1.21** ^**a**^ **(1.15, 1.3)**	42.8 %	**1.23** ^**a**^ **(1.17, 1.29)**
	**Social deprivation**							
	I (Least deprived)	6390	11.3 %	Ref	20.9 %	Ref	37.2 %	Ref
	II	6803	11.3 %	1.01 (0.91, 1.13)	21.7 %	**1.09 (1.00, 1.19)**	39.1 %	**1.13** ^**a**^ **(1.05, 1.22)**
	III	8078	12.2 %	**1.12 (1.01, 1.24)**	22.2 %	**1.12 (1.04, 1.22)**	38.7 %	**1.12 (1.04, 1.21)**
	IV	7817	11.3 %	1.05 (0.95, 1.17)	20.5 %	1.06 (0.97, 1.15)	37.5 %	**1.12 (1.04, 1.20)**
	V (Most deprived)	7886	11.9 %	**1.17 (1.05, 1.30)**	22.0 %	**1.24** ^**a**^ **(1.14, 1.34)**	37.9 %	**1.23** ^**a**^ **(1.15, 1.33)**
**SAH**	**All cases**	1753	26.9 %	-	35.6 %	-	40.4 %	-
	**Age**							
	< 65y	1043	22.5 %	-	27.0 %	-	28.9 %	-
	65–74y	317	29.7 %	-	41.0 %	-	47.3 %	-
	75–84y	266	34.6 %	-	54.1 %	-	62.4 %	-
	85y+	127	40.2 %	-	53.5 %	-	70.5 %	-
	**Gender**							
	Male	634	26.8 %	Ref	35.6 %	Ref	40.5 %	Ref
	Female	1119	27.0 %	1.08 (0.85, 1.37)	35.6 %	1.03 (0.82, 1.29)	40.3 %	1.00 (0.80, 1.25)
	**Social deprivation**							
	I (Least deprived)	291	28.2 %	Ref	34.7 %	Ref	40.0 %	Ref
	II	318	25.8 %	0.91 (0.62, 1.34)	35.5 %	1.09 (0.75, 1.56)	39.6 %	1.02 (0.71, 1.47)
	III	366	26.0 %	0.99 (0.69, 1.44)	36.3 %	1.24 (0.88, 1.76)	41.5 %	1.25 (0.88, 1.77)
	IV	355	27.0 %	1.10 (0.76, 1.59)	34.9 %	1.19 (0.83, 1.69)	39.4 %	1.13 (0.80, 1.61)
	V (Most deprived)	369	27.4 %	1.09 (0.75, 1.58)	36.9 %	1.32 (0.93, 1.87)	42.5 %	1.32 (0.93, 1.88)

Ref = Reference category. Bold font denotes significance at the 5 % level

Gender for 1 case was not recorded for AMI. No WIMD score was available for 3.2 % of AMI cases, 2.4 % of stroke cases or 3.1 % of SAH cases

^a^ denotes significance after applying a Bonferroni correction

^b^ The OR for sex gender is adjusted for age group and comorbidities. All other factors were adjusted for age group, gender and comorbidities

Social deprivation was significantly associated with higher mortality at 30 days for AMI (deprivation quintile V = 25 % (95 % CI = 12 %, 40 %, when compared with quintile I), stroke (V = 24 %; 14 %, 34 %; III = 12 %; 4 %, 22 % and II = 9 %; 0 %, 19 %) and there was a non-significant increase for SAH (V = 32 %; −7 %, 87 % - see Table 1). There was also a significant association between social deprivation and mortality at 7 days for AMI (deprivation quintile IV = 23 %; 7 %, 40 % and V = 34 %; 17 %, 53 %), stroke (V = 17 %; 5 %, 30 %) and there was a non-significant increase for SAH (V = 9 %; −25 %, 58 % - see Table 1).

Table 2 shows the numbers of admissions and 30 day mortality rates for each condition and the least and most deprived quintiles (I and V) of patients according to each study each risk factors. For the majority of factors there was a higher 30 day mortality rate for the most deprived quintile compared to the most affluent quintile. The only exceptions were dementia, public holidays, winter and spring admissions and admissions to medium sized hospitals in AMI cases, COPD, diabetes, renal failure, male gender and community hospitals in stroke cases and asthma, diabetes, dementia, female gender, spring admissions and admissions during 2004–05 and 2009–11 for SAH. The data for all five deprivation quintiles can be found as an Additional file 1.

**Table 2:** Admissions and 30 day mortality rates split by risk factors and social deprivation quintiles

Risk factors	AMI		Stroke		SAH	
	Admissions, % 30 days mortality		Admissions, % 30 days mortality		Admissions, % 30 days mortality	
	I	V	I	V	I	V
Comorbidities						
Ischaemic heart disease (I20–I25)	4776, 14.5 %	6342, 15.0 %	1873, 23.8 %	2726, 23.3 %	35, 45.7 %	83, 45.8 %
Other cardiovascular diseases (I00–I15, I26–I52)	3702, 16.0 %	4887, 16.3 %	5088, 20.3 %	6273, 21.8 %	161, 39.1 %	197, 41.1 %
Cerebrovascular disease (I60–I69)	521, 18.4 %	733, 24.0 %	6390, 20.9 %	7886, 22.0 %	291, 34.7 %	369, 36.9 %
Other circulatory diseases (I70–I99)	1391, 15.0 %	1827, 17.0 %	2289, 18.0 %	2780, 19.2 %	69, 36.2 %	88, 36.4 %
Malignancies (C00–C97)	634, 18.6 %	661, 19.5 %	986, 21.8 %	998, 24.4 %	22, 36.4 %	21, 47.6 %
Chronic obstructive pulmonary disease (J40–J44)	511, 17.6 %	1271, 18.7 %	581, 22.2 %	1433, 21.1 %	20, 40.0 %	43, 44.2 %
Asthma (J45–J46),	470, 12.3 %	864, 13.5 %	548, 17.9 %	994, 18.2 %	25, 44.0 %	53, 30.2 %
Diabetes (E10–E14)	912, 16.1 %	1492, 17.4 %	1089, 22.4 %	1857, 19.8 %	17, 52.9 %	35, 45.7 %
Dementia (F00–F03, F05.1, G30)	215, 29.8 %	318, 28.6 %	686, 25.1 %	945, 25.9 %	6, 66.7 %	12, 33.3 %
Liver disease (K70–K77)	64, 10.9 %	99, 22.2 %	101, 20.8 %	201, 23.4 %	6, 50.0 %	4, 50.0 %
Renal failure (N17–N19)	628, 25.2 %	830, 25.8 %	608, 27.3 %	824, 25.6 %	13, 61.5 %	13, 69.2 %
Age						
< 65y	1324, 3.6 %	2307, 4.9 %	871, 11.1 %	1791, 11.4 %	166, 28.3 %	228, 28.1 %
65–74y	1019, 10.2 %	1465, 13.4 %	1250, 12.8 %	1800, 15.2 %	52, 34.6 %	65, 41.5 %
75–84y	1428, 17.6 %	1576, 22.1 %	2357, 19.9 %	2587, 24.8 %	54, 50.0 %	45, 57.8 %
85 + y	1005, 28.9 %	994, 29.8 %	1912, 31.7 %	1708, 36.1 %	19, 47.4 %	31, 61.3 %
Gender						
Male	2976, 12.3 %	3680, 12.3 %	2996, 18.1 %	3761, 17.5 %	100, 30.0 %	131, 41.2 %
Female	1800, 18.2 %	2662, 18.9 %	3394, 23.4 %	4125, 26.1 %	191, 37.2 %	238, 34.5 %
Day type						
Weekdays (Mon–Fri)	3493, 14.5 %	4617, 15.4 %	4829, 20.3 %	5814, 21.7 %	213, 33.8 %	267, 33.3 %
Weekends (Sat–Sun)	1182, 13.8 %	1600, 14.1 %	1456, 22.7 %	1913, 23.0 %	74, 37.8 %	98, 45.9 %
Public holidays	101, 22.8 %	125, 14.4 %	105, 21.0 %	159, 22.0 %	4, 25.0 %	4, 50.0 %
Season						
Winter	1231, 15.5 %	1673, 15.1 %	1607, 23.1 %	1967, 23.0 %	77, 35.1 %	83, 36.1 %
Spring	1293, 16.9 %	1585, 15.0 %	1601, 20.8 %	2063, 22.4 %	70, 37.1 %	95, 31.6 %
Summer	1125, 13.1 %	1530, 15.0 %	1584, 19.3 %	1974, 20.4 %	70, 27.1 %	100, 38.0 %
Autumn	1127, 12.2 %	1554, 15.1 %	1598, 20.3 %	1882, 22.3 %	74, 39.2 %	91, 41.8 %
Year group						
2004–2005	1382, 16.4 %	1838, 16.9 %	1562, 20.8 %	2062, 23.3 %	69, 40.6 %	114, 33.3 %
2006–2008	1798, 15.2 %	2432, 15.1 %	2353, 22.2 %	3006, 22.6 %	112, 29.5 %	132, 45.5 %
2009–2011	1596, 12.1 %	2072, 13.4 %	2475, 19.7 %	2818, 20.5 %	110, 36.4 %	123, 30.9 %
Hospital size						
Small (100–399 beds)	277, 11.9 %	853, 16.5 %	288, 19.4 %	859, 21.9 %	10, 30.0 %	27, 44.4 %
Medium (400–599 beds)	2133, 15.1 %	3400, 14.3 %	2961, 21.3 %	4271, 22.9 %	102, 39.2 %	184, 38.6 %
Large (600+ beds)	2102, 14.4 %	1867, 15.4 %	2772, 21.7 %	2335, 22.4 %	173, 33.5 %	147, 36.1 %
Community hospitals	264, 14.0 %	222, 17.1 %	369, 12.2 %	421, 11.2 %	6, 0.0 %	11, 0.0 %

### Effect of risk factors on the relationship between mortality and social deprivation

#### Patient demographics

The higher mortality among the most deprived quintile of patients V, compared with the least deprived quintile I, was most pronounced among patients aged “75–84y” admitted for AMI (32 %; 9 %, 59 %), and stroke (30 %; 13 %, 50 %), and among the 85y + group for SAH (138 %; −49 %, 1004 % - see Table 3). However, when interaction effects on mortality between social deprivation and age group were assessed, they were significant only for AMI (*p* = 0.035).

**Table 3:** Mortality rates and adjusted odds ratios at 30 days following hospitalisation for social deprivation quintiles I and V, 2004 to 2012

Risk factor	AMI			Stroke			SAH		
	Mortality rate (Quintile I)	Mortality rate (Quintile V)	30 days mortality OR (95 % CI) ^b^	Mortality rate (Quintile I)	Mortality rate (Quintile V)	30 days mortality OR (95 % CI) ^b^	Mortality rate (Quintile I)	Mortality rate (Quintile V)	30 days mortality OR (95 % CI) ^b^
**Age**									
< 65y	3.6 %	4.9 %	1.35 (0.94, 1.94)	11.1 %	11.4 %	1.14 (0.88, 1.49)	28.3 %	28.1 %	1.20 (0.72, 2.02)
65–74y	10.2 %	13.4 %	1.28 (0.98, 1.67)	12.8 %	15.2 %	1.22 (0.98, 1.51)	34.6 %	41.5 %	2.03 (0.80, 5.13)
75–84y	17.6 %	22.1 %	**1.32** ^**a**^ **(1.09, 1.59)**	19.9 %	24.8 %	**1.30** ^**a**^ **(1.13, 1.50)**	50.0 %	57.8 %	1.33 (0.53, 3.38)
85+ y	28.9 %	29.8 %	1.06 (0.87, 1.29	31.7 %	36.1 %	**1.19 (1.03, 1.37)**	47.4 %	61.3 %	2.38 (0.51, 11.04)
**Gender**									
Male	12.3 %	12.2 %	**1.21 (1.03, 1.42)**	18.1 %	17.5 %	1.11 (0.97, 1.27)	30.0 %	41.2 %	**2.45 (1.21, 4.95)**
Female	18.2 %	18.9 %	**1.26 (1.07, 1.49)**	23.4 %	26.1 %	**1.36** ^**a**^ **(1.21, 1.52)**	37.2 %	34.5 %	1.04 (0.66, 1.65)
**Day of the week**									
Weekdays (Mon–Fri)	14.5 %	15.4 %	**1.27** ^**a**^ **(1.11, 1.45)**	20.3 %	21.7 %	**1.27** ^**a**^ **(1.15, 1.40)**	33.8 %	33.3 %	1.18 (0.77, 1.82)
Weekends (Sat–Sun)	13.8 %	14.1 %	1.21 (0.96, 1.54)	22.7 %	23.0 %	1.16 (0.98, 1.38)	37.8 %	45.9 %	1.96 (0.86, 4.51)
Public holidays	22.8 %	14.4 %	0.72 (0.32, 1.63)	21.0 %	22.0 %	1.15 (0.59, 2.23)	25.0 %	50.0 %	NA
**Season of admission**									
Winter	15.5 %	15.1 %	1.09 (0.87, 1.36)	23.1 %	23.0 %	1.10 (0.93, 1.30)	35.1 %	36.1 %	0.98 (0.42, 2.28)
Spring	16.9 %	15.0 %	1.09 (0.87, 1.37)	20.8 %	22.4 %	**1.29 (1.09, 1.53)**	37.1 %	31.6 %	0.99 (0.43, 2.29)
Summer	13.1 %	15.0 %	**1.36 (1.07, 1.72)**	19.3 %	20.4 %	**1.27 (1.06, 1.51)**	27.1 %	38.0 %	2.26 (0.97, 5.24)
Autumn	12.2 %	15.1 %	**1.57** ^**a**^ **(1.23, 2.01)**	20.3 %	22.3 %	**1.36** ^**a**^ **(1.14, 1.62)**	39.2 %	41.8 %	1.76 (0.75, 4.09)
**Year of admission**									
2004–2005	16.4 %	16.9 %	**1.25 (1.02, 1.53)**	20.8 %	23.3 %	**1.31** ^**a**^ **(1.10, 1.55)**	40.6 %	33.3 %	0.67 (0.27, 1.65)
2006–2008	15.2 %	15.1 %	1.16 (0.96, 1.39)	22.2 %	22.6 %	**1.23** ^**a**^ **(1.07, 1.41)**	29.5 %	45.5 %	**3.54** ^**a**^ **(1.77, 7.07)**
2009–2011	12.1 %	13.4 %	**1.32 (1.07, 1.63)**	19.7 %	20.5 %	**1.21 (1.05, 1.40)**	36.4 %	30.9 %	0.75 (0.40, 1.43)
**Hospital size**									
Small (100–399 beds)	11.9 %	16.5 %	1.50 (0.96, 2.36)	19.4 %	21.9 %	1.27 (0.89, 1.82)	30.0 %	44.4 %	NA
Medium (400–599 beds)	15.1 %	14.3 %	1.11 (0.94, 1.31)	21.3 %	22.9 %	**1.25** ^**a**^ **(1.11, 1.42)**	39.2 %	38.6 %	1.28 (0.72, 2.29)
Large (600+ beds)	14.2 %	15.4 %	**1.28 (1.06, 1.55)**	21.7 %	22.4 %	**1.20 (1.04, 1.39)**	33.5 %	36.1 %	1.44 (0.84, 2.47)
Community hospitals	14.0 %	17.1 %	1.57 (0.92, 2.69)	12.2 %	11.2 %	1.10 (0.69, 1.76)	0.0 %	0.0 %	NA

Quintile I was used as the reference group. The ORs of quintile V are reported in the table

Bold font denotes significance at the 5 % level

NA The sample size was too small to perform logistic regression

^a^ denotes significance after applying a Bonferroni correction

^b^ The OR for age group was adjusted for gender and comorbidities; gender was adjusted for age group and comorbidities; all other factors were adjusted for age group, gender and comorbidities

Mortality for deprivation quintile V compared with I was highest among females for AMI (26 %; 7 %, 49 %) and stroke (36 %; 21 %, 52 %) and among males for AMI (21 %; 3 %, 42 %) and SAH (145 %; 21 %, 395 % - see Table 3) but there were no significant interactions between gender and deprivation on mortality.

#### Timing of admission

Although mortality for deprivation quintile V versus I was highest for weekday admissions for AMI (27 %; 11 %, 45 %) and stroke (27 %; 15 %, 40 %), there were no significant interactions on mortality between week day of admission and social deprivation for SAH. Deprivation inequalities in mortality were highest for admissions during the autumn for both AMI (57 %; 23 %, 101 %) and stroke (36 %; 14 %, 62 % - see Table 3) but there was no significant variation in mortality across seasons.

When analysing deprivation-related mortality over time, there was no clear trend for AMI, with inequalities in 2004–2005 and 2009–2011 of 25 % (2 %, 53 %) and 32 % (7 %, 63 %) respectively; see Table 3), or for stroke (31 %; 10 %, 55 % in 2004–2005 and 21 %;5 %, 40 % in 2009–2011). For SAH, there was significantly higher mortality in deprived cases for 2006–2008 (3.54 fold) which was significant (*p* = 0.036).

#### Hospital size

There was no significant variation in social inequalities in mortality among quintile V versus I according to the size of the admitting hospital for any of the three conditions (Table 3).

### First admissions and their effect on mortality with social deprivation

When confining the study to first admissions for AMI, stroke and SAH during the study period, we excluded 1.3 % of AMI cases, 3.8 % of stroke cases and 0.3 % of SAH cases. The mortality rates at 30 days were unchanged at 14.3, 21.4 and 35.6 % respectively and for 7 days were marginally lower at 9.1, 11.5 and 26.9 %. There were no changes to the significance of the interaction effects on mortality between social deprivation and any of the study factors.

## Discussion

We found that social deprivation was significantly associated with higher mortality following admissions for AMI and stroke and, although not significantly, for SAH. We also found that the higher mortality at 30 days with increased social deprivation varied significantly according to patient age for AMI patients and time period for SAH. It was also highest for both AMI and stroke patients, although not significantly, for female patients, for admissions on weekdays and during autumn months.

Major strengths of the study are its size, covering more than 30,000 cases of AMI, almost 38,000 cases of stroke and over 1700 cases of SAH. The methodology was based on systematic, validated record linkage of inpatient, death certificate and primary care data to identify all admissions and all deaths that occur during the impatient stay and following discharge from hospital.

As with other large-scale studies that used NHS administrative health data, this study lacked detailed information about stroke severity, patient disease history, treatment allocated and time to treatment. Our study investigated post-hospital mortality for AMI, stroke and SAH, so did not include deaths that occurred rapidly before admission to hospital.

Our 30 day mortality rates were comparable with those reported by other NHS-based studies for AMI [28–30], stroke [1, 31, 32] and SAH [31–33]. The significant association between 30 day mortality and deprivation were also consistent with other similar studies of these conditions in the UK [2, 3, 6–8, 22, 23, 34, 35].

Although not statistically significant we found indications of increased social inequalities in mortality among older age groups, which has been reported from a study of AMI patients aged over 65y in Scotland [36], whilst another study based in Wales reported no association between deprivation and mortality for males aged 50–59y [3], supportive of our study findings for our male group.

We found highest deprivation-related mortality for admissions in autumn for AMI and stroke and in summer for SAH. However, there was no significant overall interaction effect between season and social deprivation on mortality.

Our study found no significant change over time in deprivation-related mortality for AMI and stroke from 2004–05 to 2009–11 as most of the fixed year groups analysed showed increased mortality in deprived groups. For AMI the difference was 25 % in 2004–05 and 32 % in 2009–11, and for stroke it was 31 and 21 %. There was a significantly higher, social inequality in mortality for SAH for 2006–2008 than in the other year groups.

Previous studies have reported a reduced likelihood of receiving appropriate, timely treatment for more deprived cases, and therefore, poorer outcomes for stroke [37] and cardiovascular conditions [38, 39], particularly if patients are older [40]. Deprived patients are also known to be less likely to attend or complete rehabilitation following cardiovascular conditions [40–42], which may negatively impact on outcomes.

Admissions to small hospitals showed the highest social inequality in mortality although this was not significant. This finding is supported by an American study comparing mortality rates in large and small hospitals [43]. In some cases the most severe cases are triaged or transferred to the largest hospitals, which increases mortality risks in these hospitals. However, hospital size should be regarded only as a possible indicator, since there were only four ‘large’ hospitals in our study population.

When considering the findings of this study it is important to remember that any association between (postcode-based) deprivation and higher mortality can be subject to ecological fallacy - not everyone living in a deprived area is deprived - and that not all deprived people live in deprived areas. Our measure of area social deprivation is a mixed exposure that due to economic segregation is correlated with both individual and area characteristics. Social deprivation refers to problems caused by a general lack of resources and opportunities and not just money. The association between social deprivation and higher mortality is also multifaceted. Patient-based factors could include the person’s health and wellbeing, with deprived people more likely to have single and multiple comorbidities compared with more affluent patients [44], reduced resources (car and home ownership), lower education status and higher unemployment. Lower social class is associated with lifestyle factors such as smoking and poorer mental health [45] which can also affect prognosis following AMI, stroke and SAH. As such, a patient’s baseline clinical and psychological status may largely contribute to any relationship between social deprivation and mortality. We found that the influence of patient demographics, timing of admission and hospital size on social inequalities in mortality are quite similar for AMI and stroke.

## Conclusions

To conclude, we have demonstrated a clear association between social deprivation and higher mortality following emergency admission for AMI, stroke and SAH. The study findings also suggest that the influence of patient demographics, timing of admission and hospital size on social deprivation inequalities in mortality are quite similar for AMI and stroke.

## Additional file

Additional file 1:
**Extended Table 2 showing the number of admissions and 30d mortality rates for all five deprivation quintiles.**ᅟ

## Abbreviations

ALF: Anonymised linking field
AMI: Acute myocardial infarction
AR: Administrative Register
DGH: District General Hospital
ICD-10: International Classification of Diseases tenth revision
MACRAL: Matching Algorithm for Consistent Results in Anonymised Linkage
NHS: National Health Service
ONS: Office of National Statistics
PEDW: Patient Episode Database for Wales
SAH: Subarachnoid haemorrhage
SAIL: Secure Anonymised Information Linkage
WIMD: Welsh Index of Multiple Deprivation
