# Supplementary material for: The impact of social deprivation on mortality following acute myocardial infarction, stroke or subarachnoid haemorrhage: A record linkage study
Source: BMC Cardiovasc Disord. 2015 Jul 18;15:71. doi: 10.1186/s12872-015-0045-x (PMC4506594; doi:10.1186/s12872-015-0045-x)
Supplement: Additional file 1: — Extended Table 2 showing the number of admissions and 30d mortality rates for all five deprivation quintiles.ᅟ [file 12872_2015_45_MOESM1_ESM.docx]

|  |  |  |  |
| --- | --- | --- | --- |

|  | **Risk factors** | **Quintile I**  **Admissions,**  **% 30d mortality** | **Quintile QII**  **Admissions,**  **% 30d mortality** | **Quintile III**  **Admissions,**  **% 30d mortality** | **Quintile IV**  **Admissions,**  **% 30d mortality** | **Quintile V**  **Admissions,**  **% 30d mortality** |
| --- | --- | --- | --- | --- | --- | --- |
| AMI | **Comorbidities** Ischaemic heart disease (I20-I25) | 4776, 14.5% | 5527, 13.4% | 6579, 14.3% | 6460, 14.3% | 6342, 15.0% |
|  | Other cardiovascular diseases (I00-I15, I26-I52) | 3702, 16.0% | 4264, 14.9% | 5157, 15.5% | 5058, 15.9% | 4887, 16.3% |
|  | Cerebrovascular disease (I60-I69) | 521, 18.4% | 531, 18.6% | 680, 22.2% | 750, 22.9% | 733, 24.0% |
|  | Other circulatory diseases (I70-I99) | 1391, 15.0% | 1565, 15.7% | 1980, 16.0% | 1881, 16.7% | 1827, 17.0% |
|  | Malignancies (C00-C97) | 634, 18.6% | 625, 17.9% | 772, 18.1% | 726, 18.3% | 661, 19.5% |
|  | Chronic obstructive pulmonary disease (J40-J44) | 511, 17.6% | 684, 18.1% | 968, 15.9% | 1120, 17.9% | 1271, 18.7% |
|  | Asthma (J45-J46), | 470, 12.3% | 567, 11.6% | 748, 11.9% | 801, 12.2% | 864, 13.5% |
|  | Diabetes (E10-E14) | 912, 16.1% | 1053, 16.9% | 1390, 17.1% | 1384, 17.1% | 1492, 17.4% |
|  | Dementia (F00-F03, F05.1, G30) | 215, 29.8% | 287, 24.4% | 299, 29.8% | 306, 33.3% | 318, 28.6% |
|  | Liver disease (K70-K77) | 64, 10.9% | 83, 24.1% | 85, 17.6% | 92, 22.8% | 99, 22.2% |
|  | Renal failure (N17-N19) | 628, 25.2% | 644, 28.1% | 878, 27.3% | 846, 25.9% | 830, 25.8% |
|  | **Age** <65y | 1324, 3.6% | 1629, 3.1% | 2048, 3.6% | 2139, 4.7% | 2307, 4.9% |
|  | 65-74y | 1019, 10.2% | 1305, 8.0% | 1536, 9.9% | 1490, 12.2% | 1465, 13.4% |
|  | 75-84y | 1428, 17.6% | 1586, 17.7% | 1842, 20.1% | 1737, 20.7% | 1576, 22.1% |
|  | 85+y | 1005, 28.9% | 1007, 30.4% | 1153, 29.7% | 1094, 29.8% | 994, 29.8% |
|  | **Gender** Male | 2976, 12.3% | 3357, 11.4% | 3997, 12.0% | 3766, 12.6% | 3680, 12.3% |
|  | Female | 1800, 18.2% | 2170, 16.5% | 2582, 17.9% | 2693, 18.4% | 2662, 18.9% |
|  | **Day type** Weekdays (Mon- Fri) | 3493, 14.5% | 4037, 13.4% | 4760, 14.0% | 4647, 14.8% | 4617, 15.4% |
|  | Weekends (Sat - Sun) | 1182, 13.8% | 1382, 13.5% | 1693, 14.6% | 1655, 15.6% | 1600, 14.1% |
|  | Public holidays | 101, 22.8% | 108, 13.9% | 126, 19.0% | 158, 14.6% | 125, 14.4% |
|  | **Season** Winter | 1231, 15.5% | 1443, 13.0% | 1690, 15.6% | 1602, 15.7% | 1673, 15.1% |
|  | Spring | 1293, 16.9% | 1491, 13.8% | 1729, 13.9% | 1699, 14.7% | 1585, 15.0% |
|  | Summer | 1125, 13.1% | 1290, 12.9% | 1597, 13.0% | 1543, 14.5% | 1530, 15.0% |
|  | Autumn | 1127, 12.2% | 1303, 13.9% | 1563, 14.6% | 1616, 15.0% | 1554, 15.1% |
|  | **Year group** 2004 – 2005 | 1382, 16.4% | 1483, 15.4% | 1836, 15.3% | 1826, 17.7% | 1838, 16.9% |
|  | 2006 – 2008 | 1798, 15.2% | 2150, 14.4% | 2523, 15.8% | 2464, 14.1% | 2432, 15.1% |
|  | 2009 – 2011 | 1596, 12.1% | 1894, 10.8% | 2220, 14.3% | 2170, 13.7% | 2072, 13.4% |
|  | **Hospital size** Small (100-399 beds) | 277, 11.9% | 1548, 12.3% | 2087, 13.5% | 1233, 16.4% | 853, 16.5% |
|  | Medium (400-599 beds) | 2133, 15.1% | 2083, 13.2% | 2645, 14.1% | 2880, 14.5% | 3400, 14.3% |
|  | Large (600+ beds) | 2102, 14.4% | 16.8, 13.9% | 1530, 15.0% | 1969, 14.6% | 1867, 15.4% |
|  | Community hospitals | 264, 14.0% | 288, 18.1% | 317, 17.0% | 388, 16.0% | 222, 17.1% |
| Stroke | **Comorbidities** Ischaemic heart disease (I20-I25) | 1873, 23.8% | 2083, 23.5% | 2633, 24.6% | 2564, 22.5% | 2726, 23.3% |
|  | Other cardiovascular diseases (I00-I15, I26-I52) | 5088, 20.3% | 5463, 21.6% | 6435, 22.0% | 6271, 20.9% | 6273, 21.8% |
|  | Cerebrovascular disease (I60-I69) | 6390, 20.9% | 6803, 21.7% | 8078, 22.2% | 7817, 20.5% | 7886, 22.0% |
|  | Other circulatory diseases (I70-I99) | 2289, 18.0% | 2290, 18.8% | 2978, 18.0% | 2783, 17.6% | 2780, 19.2% |
|  | Malignancies (C00-C97) | 986, 21.8% | 910, 23.2% | 1150, 22.1% | 1014, 23.2% | 998, 24.4% |
|  | Chronic obstructive pulmonary disease (J40-J44) | 581, 22.2% | 737, 22.9% | 967, 23.0% | 1133, 21.3% | 1433, 21.1% |
|  | Asthma (J45-J46), | 548, 17.9% | 594, 19.0% | 752, 20.7% | 848, 17.5% | 994, 18.2% |
|  | Diabetes (E10-E14) | 1089, 22.4% | 1304, 20.9% | 1644, 21.0% | 1749, 19.6% | 1857, 19.8% |
|  | Dementia (F00-F03, F05.1, G30) | 686, 25.1% | 818, 28.0% | 963, 25.1% | 980, 24.6% | 945, 25.9% |
|  | Liver disease (K70-K77) | 101, 20.8% | 107, 18.7% | 130, 14.6% | 155, 15.5% | 201, 23.4% |
|  | Renal failure (N17-N19) | 608, 27.3% | 680, 26.6% | 814, 27.5% | 800, 24.6% | 824, 25.6% |
|  | **Age** <65y | 871, 11.1% | 1074, 11.6% | 1335, 11.3% | 1512, 9.4% | 1791, 11.4% |
|  | 65-74y | 1250, 12.8% | 1354, 16.0% | 1681, 14.9% | 1665, 15.3% | 1800, 15.2% |
|  | 75-84y | 2357, 19.9% | 2507, 20.8% | 2787, 22.1% | 2641, 20.6% | 2587, 24.8% |
|  | 85+y | 1912, 31.7% | 1868, 32.9% | 2275, 34.0% | 1999, 33.1% | 1708, 36.1% |
|  | **Gender** Male | 2996, 18.1% | 3249, 18.4% | 3763, 18.2% | 3703, 16.7% | 3761, 17.5% |
|  | Female | 3394, 23.4% | 3554, 24.8% | 4315, 25.7% | 4114, 24.0% | 4125, 26.1% |
|  | **Day type** Weekdays (Mon- Fri) | 4829, 20.3% | 5074, 20.7% | 5946, 21.6% | 5816, 19.9% | 5814, 21.7% |
|  | Weekends (Sat - Sun) | 1456, 22.7% | 1588, 24.6% | 1976, 23.6% | 1836, 21.7% | 1913, 23.0% |
|  | Public holidays | 105, 21.0% | 141, 28.4% | 156, 26.3% | 165, 27.3% | 159, 22.0% |
|  | **Season** Winter | 1607, 23.1% | 1727, 23.2% | 2024, 24.2% | 1898, 21.4% | 1967, 23.0% |
|  | Spring | 1601, 20.8% | 1694, 23.4% | 2078, 23.2% | 2027, 21.9% | 2063, 22.4% |
|  | Summer | 1584, 19.3% | 1719, 18.8% | 1981, 19.3% | 1979, 18.8% | 1974, 20.4% |
|  | Autumn | 1598, 20.3% | 1663, 21.5% | 1995, 22.0% | 1913, 20.0% | 1882, 22.3% |
|  | **Year group** 2004 – 2005 | 1562, 20.8% | 1787, 23.0% | 2058, 24.3% | 2025, 21.1% | 2062, 23.3% |
|  | 2006 – 2008 | 2353, 22.2% | 2508, 21.9% | 3019, 22.6% | 2933, 21.5% | 3006, 22.6% |
|  | 2009 – 2011 | 2475, 19.7% | 2508, 20.6% | 3001, 20.3% | 2859, 19.1% | 2818, 20.5% |
|  | **Hospital size** Small (100-399 beds) | 288, 19.4% | 1602, 21.2% | 2306, 21.6% | 1322, 20.4% | 859, 21.9% |
|  | Medium (400-599 beds) | 2961, 21.3% | 2735, 23.0% | 3328, 22.8% | 3673, 21.7% | 4271, 22.9% |
|  | Large (600+ beds) | 2772, 21.7% | 1852, 22.8% | 1853, 24.3% | 2214, 20.9% | 2335, 22.4% |
|  | Community hospitals | 369, 12.2% | 614, 14.2% | 591, 14.4% | 608, 12.3% | 421, 11.2% |
| SAH | **Comorbidities** Ischaemic heart disease (I20-I25) | 35, 45.7% | 36, 47.2% | 38, 44.7% | 54, 44.4% | 83, 45.8% |
|  | Other cardiovascular diseases (I00-I15, I26-I52) | 161, 39.1% | 164, 39.0% | 182, 39.6% | 184, 37.0% | 197, 41.1% |
|  | Cerebrovascular disease (I60-I69) | 291, 34.7% | 318, 35.5% | 366, 36.3% | 355, 34.9% | 369, 36.9% |
|  | Other circulatory diseases (I70-I99) | 69, 36.2% | 63, 38.1% | 86, 32.6% | 96, 33.3% | 88, 36.4% |
|  | Malignancies (C00-C97) | 22, 36.4% | 19, 36.8% | 26, 34.9% | 26, 46.2% | 21, 47.6% |
|  | Chronic obstructive pulmonary disease (J40-J44) | 20, 40.0% | 22, 36.4% | 25, 56.0% | 32, 43.8% | 43, 44.2% |
|  | Asthma (J45-J46), | 25, 44.0% | 38, 26.3% | 35, 54.3% | 28, 32.1% | 53, 30.2% |
|  | Diabetes (E10-E14) | 17, 52.9% | 12, 25.0% | 26, 57.7% | 27, 40.7% | 35, 45.7% |
|  | Dementia (F00-F03, F05.1, G30) | 6, 66.7% | 15, 26.7% | 14, 21.4% | 9, 33.3% | 12, 33.3% |
|  | Liver disease (K70-K77) | 6, 50.0% | 6, 33.3% | 7, 42.9% | 10, 30.0% | 4, 50.0% |
|  | Renal failure (N17-N19) | 13, 61.5% | 7, 14.3% | 14, 50.0% | 15, 33.3% | 13, 69.2% |
|  | **Age** <65y | 166, 28.3% | 178, 23.0% | 218, 26.6% | 214, 29.0% | 228, 28.1% |
|  | 65-74y | 52, 34.6% | 58, 44.8% | 70, 52.9% | 61, 29.5% | 65, 41.5% |
|  | 75-84y | 54, 50.0% | 57, 54.4% | 52, 57.7% | 54, 50.0% | 45, 57.8% |
|  | 85+y | 19, 47.4% | 25, 60.0% | 26, 30.8% | 26, 65.4% | 31, 61.3% |
|  | **Gender** Male | 100, 30.0% | 116, 35.3% | 135, 38.5% | 128, 32.8% | 131, 41.2% |
|  | Female | 191, 37.2% | 202, 35.6% | 231, 35.1% | 227, 36.1% | 238, 34.5% |
|  | **Day type** Weekdays (Mon- Fri) | 213, 33.8% | 231, 34.2**%** | 265, 37.0% | 270, 37.8% | 267, 33.3% |
|  | Weekends (Sat - Sun) | 74, 37.8% | 85, 38.8% | 96, 34.4% | 77, 26.0% | 98, 45.9% |
|  | Public holidays | 4, 25.0% | 2, 50.0% | 5, 40.0% | 8, 25.0% | 4, 50.0% |
|  | **Season** Winter | 77, 35.1% | 92, 33.7% | 82, 36.6% | 78, 32.1% | 83, 36.1% |
|  | Spring | 70, 37.1% | 73, 38.4% | 86, 34.9% | 84, 33.3% | 95, 31.6% |
|  | Summer | 70, 27.1% | 86, 37.2% | 92, 39.1% | 99, 34.3% | 100, 38.0% |
|  | Autumn | 74, 39.2% | 67, 32.8% | 106, 34.9% | 94, 39.4% | 91, 41.8% |
|  | **Year group** 2004 – 2005 | 69, 40.6% | 95, 35.8% | 114, 38.6% | 105, 32.4% | 114, 33.3% |
|  | 2006 – 2008 | 112, 29.5% | 112, 33.0% | 127, 33.1% | 120, 35.0% | 132, 45.5% |
|  | 2009 – 2011 | 110, 36.4% | 111, 37.8% | 125, 37.6% | 130, 36.9% | 123, 30.9% |
|  | **Hospital size** Small (100-399 beds) | 10, 30.0% | 65, 38.5% | 92, 37.0% | 50, 40.0% | 27, 44.4% |
|  | Medium (400-599 beds) | 102, 39.2% | 109, 31.2% | 150, 38.0% | 156, 37.2% | 184, 38.6% |
|  | Large (600+ beds) | 173, 33.5% | 133, 39.1% | 116, 36.2% | 138, 32.6% | 147, 36.1% |
|  | Community hospitals | 6, 0.0% | 11, 18.2% | 8, 0.0% | 11, 9.1% | 11, 0.0% |
